# 6-Formylindolo(3,2-b)Carbazole (FICZ) Modulates the Signalsome Responsible for RA-Induced Differentiation of HL-60 Myeloblastic Leukemia Cells

**DOI:** 10.1371/journal.pone.0135668

**Published:** 2015-08-19

**Authors:** Rodica P. Bunaciu, Holly A. Jensen, Robert J. MacDonald, Dorian H. LaTocha, Jeffrey D. Varner, Andrew Yen

**Affiliations:** 1 Department of Biomedical Sciences, Cornell University, Ithaca, New York, 14853, United States of America; 2 School of Chemical and Biomolecular Engineering, Cornell University, Ithaca, New York, 14853, United States of America; 3 Flow Cytometry Core Facility, Cornell University, Ithaca, New York, 14853, United States of America; The Ohio State University, UNITED STATES

## Abstract

6-Formylindolo(3,2-b)carbazole (FICZ) is a photoproduct of tryptophan and an endogenous high affinity ligand for aryl hydrocarbon receptor (AhR). It was previously reported that, in patient-derived HL-60 myeloblastic leukemia cells, retinoic acid (RA)-induced differentiation is driven by a signalsome containing c-Cbl and AhR. FICZ enhances RA-induced differentiation, assessed by expression of the membrane differentiation markers CD38 and CD11b, cell cycle arrest and the functional differentiation marker, inducible oxidative metabolism. Moreover, FICZ augments the expression of a number of the members of the RA-induced signalsome, such as c-Cbl, Vav1, Slp76, PI3K, and the Src family kinases Fgr and Lyn. Pursuing the molecular signaling responsible for RA-induced differentiation, we characterized, using FRET and clustering analysis, associations of key molecules thought to drive differentiation. Here we report that, assayed by FRET, AhR interacts with c-Cbl upon FICZ plus RA-induced differentiation, whereas AhR constitutively interacts with Cbl-b. Moreover, correlation analysis based on the flow cytometric assessment of differentiation markers and western blot detection of signaling factors reveal that Cbl-b, p-p38α and pT390-GSK3β, are not correlated with other known RA-induced signaling components or with a phenotypic outcome. We note that FICZ plus RA elicited signaling responses that were not typical of RA alone, but may represent alternative differentiation-driving pathways. In clusters of signaling molecules seminal to cell differentiation, FICZ co-administered with RA augments type and intensity of the dynamic changes induced by RA. Our data suggest relevance for FICZ in differentiation-induction therapy. The mechanism of action includes modulation of a SFK and MAPK centered signalsome and c-Cbl-AhR association.

## Introduction

Retinoic acid (RA), a metabolite of vitamin A, is an important developmental morphogen with pleiotropic actions. The most studied RA developmental effects are the specification of the anterior- posterior axis and left–right patterning [1]. RA, through its signaling and downstream transcriptional targets, regulates the differentiation, development and functions of hematopoietic cells and particularly myeloid and lymphocytic progenitors. In the bone marrow cellular compartment, RA normally promotes granulocytic development to the detriment of erythroid [2] and myeloid dendritic cell differentiation [3]. One of the most prominent effects of RA on hematopoiesis is in the neutrophilic series, both in normal granulopoiesis and especially in acute promyelocytic leukemia (APL) differentiation therapy. APL is a subtype of the acute myeloid leukemia and is classified as FAB M3. RA induces remission in almost all APL PML/RARalpha+ patients [4, 5]. However, the remission is not durable and the relapsed cases are resistant to retinoid treatment [6]. To reduce potential relapse, combination therapy of RA and chemotherapy is used [6]. A recent study, analyzing the dataset derived from the North American Intergroup Study INT0129, calculated for the first time the estimated duration of RA needed after chemotherapy to eliminate the leukemic stem cell population to be one year [7]. This study showed that RA can eliminate the cancer stem cell population by inducing differentiation of the blasts and modulating the cell cycle of the cancer stem cells. Prior to this, it was thought that RA could overcome a block in differentiation but did not eliminate the leukemic clone. In patients with relapsed APL, RA plus arsenic trioxide was found to be effective [8]. Some experts therefore advocate a front line therapy of RA and arsenic trioxide without chemotherapy from the initial diagnosis for patients with low to intermediate risk APL [9–11]. In elderly APL patients, or patients not qualifying for chemotherapy or arsenic trioxide therapy due to concurrent disease, RA as a single therapy was reported to be effective in both induction and maintenance of remission [12, 13]. Clinically achievable plasma concentrations reach 1 μM, which is the concentration typically used in *in vitro* studies [14]. Currently, the use of retinoid treatment is being expanded to other AMLs (NCT01237808, NCT00892190, NCT00867672, NCT00995332, NCT02261779, NCT00326170) and even other pathologies (NCT00062010, NCT02173054), as reviewed in [15]. Finally there is data suggesting that RA-therapy, used as part of a combination therapy, can be extended beyond leukemias to other tumors. Recently Retinoic Acid-Induced 2 protein (RAI2) was proposed as a metastasis suppressor [16]. In lung cancer treatment and prevention, vitamin A was shown to be detrimental to high risk patients (smokers and asbestos workers) [17]. However, a very recent study shows a crucial benefit of RA pretreatment prior to cisplatin treatment for non-small-cell lung cancer. RA pretreatment counteracts cisplatin resistance by inducing differentiation of the slow dividing tumor initiating cells CD133+/CXCR4+ (multipotent progenitor) cells [18]. Therapies that combine retinoids and other modalities are very diverse and used both for combined targeting of multiple pathways and for diminishing toxicity, but mechanistic insights are needed for their improved design.

A much used experimental model for RA response of a non-APL myeloid leukemia is the HL-60 cell line. Bipotent human acute myelogenous leukemia HL-60 cells (an NCI-60 cell line) have a high proliferative rate in culture and do not bear the t(15,17) translocation that defines RA-responsive APL, yet they are RA-responsive. This makes HL-60 an attractive model for response in a non-APL myeloid leukemia. Studying the RA-dependent pathways that are independent of t(15,17) are crucial to expanding the efficacy of RA as a therapeutic agent. Generally, the mechanism of action of RA can be categorized as nuclear and extra-nuclear. Furthermore, nuclear effects can be categorized as transcriptional or epigenetic. Extra-nuclear effects can be described as plasma membrane-associated or cytosolic [15]. In HL-60 cells, RA causes G_0_/G_1_ arrest and myeloid differentiation characterized by upregulated expression of membrane-bound CD38 and CD11b and increased inducible oxidative metabolism. We and others have shown that this differentiation is driven by a signalsome that includes an ensemble of signaling molecules that are known to mediate numerous pathways. This process requires sustained activation of MAPK signaling [19–21], and involves regulatory signaling events including the Src family kinases Fgr and Lyn, PI3K, c-Cbl, Slp76, Vav1, CK2, and KSR, as well as the IRF-1 transcription factor [22–26]. These factors display RA-upregulated expression. The ability to drive differentiation has been directly demonstrated for a number of these putative signalsome components. For example, during RA-induced differentiation, ectopic expression of c-Raf [27, 28], c-Cbl [23], Slp76 partnered with c-FMS [24], IRF-1 [25] and AhR [29] have been shown to enhance MAPK signal activation and promote RA-induced differentiation and G_0_/G_1_ arrest. One of the earliest cell surface differentiation markers in HL-60 upon RA treatment is CD38, a transmembrane protein that has a significant role in propelling the differentiation process [23, 28, 30–34]. CD38 is highly expressed in granulocytes [35] and is transcriptionally regulated by RARα via a RARE in the first intron [36, 37]. Myeloid precursors ectopically overexpressing CD38 show an enhanced rate of differentiation indicated by increased inducible oxidative metabolism by 48 hours and G_0_/G_1_ arrest by 72 hours [38], and RNAi directed toward CD38 impairs RA-induced differentiation [39]. The CD38 cytoplasmic domain is associated with multiple proteins, one of which is the E3 ubiquitin ligase and adaptor c-Cbl [23]. When HL-60 cells arrest, multiple tyrosine phosphorylation events occur as part of the transmembrane signal transduction. One of these tyrosine phosphorylation events is the phosphorylation of c-Cbl [40]. Moreover, overexpression of c-Cbl augments basal CD38 expression and propels RA-induced differentiation and MAPK activation, whereas c-Cbl knockdown blunts differentiation [23]. The c-Cbl tyrosine kinase binding domain mutant (G306E) is unable to associate with CD38 and drive MAPK signaling and differentiation. c-Cbl is ergo an important effector for driving CD38 signaling during RA-induced differentiation. Consistent with a regulatory role, c-Cbl mutation has pathological significance. Cbl syndrome is often associated with juvenile myelomonocytic leukemia, a disease characterized by overproduction of monocytes [41, 42]. Interestingly, juvenile myelomonocytic leukemia is responsive to retinoid treatment [43–45].

The aryl hydrocarbon receptor (AhR) is a component of the signaling machine and a known environmental sensor, making it suspect as a regulator thereof. Indeed it has already been well implicated in developmental processes, as well as connected to the action of RA. We and others [29, 46] have shown that RA can upregulate expression of AhR. RA in excess and the AhR exogenous ligand TCDD are teratogens and each is able to induce cleft palate [47–51]. AhR ligands like dioxins, even when present at low concentrations, might promote cleft palate formation by modifying the kinetics of this multifactorial pathological process [52]. In mice, RA activates RARγ to upregulate AhR expression in the nasal mesenchyme [46]. As RA and AhR can have related pathology, and as FICZ, an endogenous AhR ligand upregulates RA-induced differentiation to neutrophils [53]—another cell type of mesenchymal origin- we investigated the mechanistic details of FICZ augmented RA-induced differentiation to neutrophils.

We previously reported that FICZ is capable of augmenting RA-induced expression of signalsome components such as AhR, c-Cbl, Vav1, Slp76, PI3K, Fgr and Lyn, as well as the conversion to a differentiated phenotype [53]. Here we report that in the FICZ plus RA-induced signalsome, there is an RA-dependent association of AhR with c-Cbl and a constitutive association with Cbl-b. By clustering analysis based on protein expression and phosphorylation data, we show that FICZ added to RA treatment both elicits new pathways and augments some of the RA-induced pathways. This provides mechanistic insight for potentially expanding the efficacy of retinoid therapy to contexts currently only marginally responsive to it by adding FICZ. Arguably one of the most striking insights emerging here is that a ligand activated transcription factor is in a cytosolic signaling machine and in fact can regulate signaling intensity to drive likely both transcriptional and cytosolic events and eventually differentiation of a leukemic stem-like cell.

## Materials and Methods

### Cell culture and treatments

HL-60 human myeloblastic leukemia cells derived from the original patient isolate, a generous gift of Dr. Robert Gallagher, were grown in RPMI 1640 (Invitrogen, Carlsbad, CA) supplemented with 5% fetal bovine serum (Hyclone, Logan,UT) and 1x antibiotic/antimycotic (Sigma, St. Louis, MO) in a 5% CO_2_ humidified atmosphere at 37°C. The cells were cultured in constant exponential growth as previously described [54]. The experimental cultures were initiated at a density of 0.1 x 10^6^ cells/ml. Viability was monitored by 0.2% trypan blue (Invitrogen, Calsbad, CA) exclusion and routinely exceeded 95%.

For treatments, all-trans-retinoic acid (RA) (Sigma, St. Louis, MO) was added from a 5 mM stock solution in 100% ethanol to a final concentration of 1 μM in culture. 6-Formylindolo(3,2-b)carbazole (FICZ) (BML-GR206-0100, Enzo Life Sciences, Exeter, United Kingdom and Abcam, Cambridge, MA ab141631), was added from a 100 μM DMSO stock to make a final concentration of 100 nM in culture. α-naphthoflavone (α-NF)and β-naphthoflavone (β-NF, both from Sigma, St. Louis, MO) were each used at a final concentration of 1 μM in culture.

### Antibodies and reagents

Antibodies for live cell cytometric analysis of CD38-PE (#555460), CD11b-PE (#555388), for CD38 (#5554580, mouse for FRET) and CD38 (#611114 mouse for WB) were from BD Pharmingen. (San Jose, CA). The c-Cbl (C-15, sc-170, rabbit for WB and FRET), AhR (H-211, sc-5578 rabbit for WB and FRET) and Cbl-b (G-1, sc-8006 mouse for FRET) were from Santa Cruz Biotechnology (Santa Cruz, CA). The AhR (RPT9, ab2769, mouse for FRET), MAFB (ab66506, rabbit, for WB), mouse monoclonal IgG1 (ab91353, for FRET) were from Abcam (Cambridge, MA). Antibodies for Cbl-b (#9498, rabbit), Lyn (#2732, rabbit), Fgr (#2755, rabbit), c-Raf (#9422, rabbit), pS259c-Raf (#9421, rabbit), pS289/296/301c-Raf (#9431, rabbit), pMEK (#9154, rabbit), pERK (#4370, rabbit), ARNT (#3414, rabbit), PU.1 (#2258, rabbit), pY416SFK (#2101, rabbit), Slp76 (#4958, rabbit), pT390GSK3β (#3548, rabbit), p(S21/9)GSK3α/β (#8566, rabbit), GSK3 (#5676, rabbit), Vav1 (#2502, rabbit), p47^phox^ (#4312, rabbit), SLP-76 (#4958, rabbit), p38α (9228, mouse), p-p38α (#9216, mouse), p-p85 PI3K (#4228, rabbit), RXRα (#5388, rabbit), GAPDH (#5174), HRP anti-mouse, and HRP anti-rabbit (for WB) and, normal rabbit polyclonal IgG (unconjugated) #2729 (for FRET),were from Cell Signaling (Danvers, MA). The pS621c-Raf (44504G) rabbit antibody (for WB) was from Invitrogen.

The goat anti-rabbit IgG (H+L) Alexa Fluor 594 conjugated (A-11037) and goat anti-mouse IgG (H+L) Alexa Fluor 488 conjugated (A-11029) were from Life Technologies (Thermo Fisher Scientific). M-PER Mammalian Protein Extraction Reagent lysis buffer was from Pierce (Rockford, IL). ECL was from GE Healthcare (Pittsburgh, PA). Propidium iodide, protease and phosphatase inhibitors, and DMSO were purchased from Sigma (St. Louis, MO).

### Cell growth and cell cycle quantification

1 x 10^6^ cells were collected by centrifugation at 700 rpm and resuspended in 200 μl of cold propidium iodide (PI) hypotonic staining solution containing 50 μg/ml propidium iodine, 1 μl/ml Triton X-100, and 1 mg/ml sodium citrate (all Sigma, St. Louis, MO). Cells were incubated at room temperature for 1 h and analyzed by flow cytometry with a BD LSRII flow cytometer (BD Biosciences, San Jose, CA) using 488-nm excitation and emission collected through 550 long-pass dichroic and a 575/26 band-pass filters. Doublets were identified by a PI signal width versus area plot and excluded from the analysis as previously described [55]. The statistical significance was determined by one way ANOVA with Tukey’s multiple comparison test using GraphPad Prism (n = 4).

### CD38 and CD11b quantification

Expression of cell surface differentiation markers was quantified by flow cytometry. 1 x 10^6^ cells were collected from cultures and centrifuged at 700 rpm for 5 min. Cell pellets were resuspended in 200 μl 37°C PBS containing 2.5 μl of phycoerythrin (PE)-conjugated CD11b or CD38 antibody (both from BD Biosciences, San Jose, CA). Following 1 h incubation at 37°C cell surface expression levels were analyzed by flow cytometry. PE was excited at 488 nm and emission was collected through 505 long-pass dichroic and 530/30 band-pass filters. Undifferentiated control cells were used to determine the fluorescence intensity of cells negative for the respective surface antigen. Area mean of the population is presented. The statistical significance was considered at p<0.05 as determined by t-test of the groups of interest (n = 3 for 48 h and n = 4 for 72 h).

### Respiratory burst quantification

1 x 10^6^ cells were collected and centrifuged at 700 rpm for 5 min. Cell pellets were resuspended in 500 μl of 2 mg/ml nitroblue tetrazolium (NBT) (Sigma) in PBS containing either 200 ng/ml 12-o-tetradecanoylphorbol-13-acetate (TPA) (Sigma) in DMSO carrier or equivalent volume of DMSO carrier alone. After incubation for 1 h at 37°C, samples were resuspended in 200 μl of 37% HCl (12 M). Absorbance was read at 595 nm. Stock NBT was maintained between 25 and 35 mg/ml in DMSO. The statistical significance was considered at p<0.05 as determined by t-test of the groups of interest (n = 4 for 48 h, n = 3 for 72 h).

### Aldehyde dehydrogenase enzymatic activity assay

ALDH1 enzymatic activity was measured using the Aldefluor kit (Stem Cell Technologies) as described by Ginestier and coworkers [56], with the modification that the cells were incubated with the substrate at 37°C for 50 min instead of 40 min. The statistical significance was considered at p<0.05 as determined by one-way ANOVA and Tukey’s multiple comparisons test using GraphPad Prism (n = 3).

### Glucose uptake assay

Glucose uptake assay was performed using the 6-NBDG (6-(N-(7-Nitrobenz-2-oxa-1,3-diazol-4-yl)amino)-6-Deoxyglucose) (from Invitrogen). The fluorochrome was excited at 488 nm and emission was collected through 505 long-pass dichroic and 530/30 band-pass filters. Area mean of each population is presented. The statistical significance was considered at p<0.05 as determined by t-test of the groups of interest (n = 4).

### Correlation and Clustering analysis

The Pearson correlation coefficient between endpoint values was calculated using the formula:

$$\rho \left(x,y\right)=\;\frac{cov\left(x,y\right)}{\sigma_{x}\sigma_{y}}=\;\frac{\sum_{i=1}^{N}\left(x_{i}-\bar{x}\right)\left(y_{i}-\bar{y}\right)}{\sqrt{\sum_{i=1}^{N}{\left(x_{i}-\bar{x}\right)}^{2}}\sqrt{\sum_{i=1}^{N}{\left(y_{i}-\bar{y}\right)}^{2}}}$$ (1)

To assess the correlation between endpoints of interest such as signaling molecule expression levels, differentiation markers, and protein-protein interactions, hierarchical clustering analysis was conducted using an average linkage method where the distance D between clusters X and Y is as follows:

$$D\left(X,Y\right)=\;\frac{1}{\left|X\right|\left|Y\right|}\sum_{x\in X}^{}\sum_{y\in Y}^{}d\left(x,y\right)$$ (2)

where the distance metric *d*(*x*, *y*) between cluster elements *x* and *y* is 1-Pearson correlation coefficient. Clustering analysis and similarity matrices based on the Pearson correlation were calculated using MATLAB software.

### RNA isolation, first strand DNA synthesis and real-time PCR

The RNA was isolated using the RNeasy Plus Mini Kit (Qiagen, Valencia, CA) according to the manufacturer’s protocol, including the use of genomic DNA eliminator columns. The SuperScript III First-Strand Synthesis System for RT-PCR (Invitrogen, Grand Island, NY) and oligo(dT)_20_ primers were used to synthesize the cDNA starting from 3 μg RNA, according to the manufacturer’s protocol, including the use of RNase after cDNA synthesis. Real-time PCR was performed in duplicates using the iQ Sybre Green Supermix kit (BioRad) on a CFX96 Touch real-time PCR detection system (BioRad). The primers used in this study were ordered from Integrated DNA Technologies: Cyp1A1: (GTA GTG CTC CTT GAC CAT CTT C and CCA GCT GAC TTC ATC CCT ATT C) and GAPDH (IDT primers only, assay ID hs.pt.39a.22214836). To verify the accuracy and specificity of each real-time PCR reading, the dissociation curves were analyzed. Data were analyzed with the ΔΔCq method and the results of three biological replicates at two treatment time points (2 h and 24 h) were compared using two-way ANOVA with Tukey’s multiple comparisons test (GraphPad Prism Software).

### Western blotting

Whole cell lysates of about 30x10^6^ HL-60 cells were prepared using 200 μL of a Pierce M-PER lysis buffer solution (Pierce, Rockford, IL) containing phosphatase and protease inhibitors (Sigma, St. Louis, MO). Lysates were centrifuged at 16000 RCF for 30 minutes. Equal amounts of protein lysates (25 μg) were resolved by gel electrophoresis and transferred onto Immobilon-P PVDF membrane (Millipore Corporation, Billerica MA). Experiments were repeated at least three times. All the western blot data were quantified using ImageJ, normalized to GAPDH and then fold-change from untreated control was calculated and graphed as min to max floating bars with a line at the mean using GraphPad Prism software. P-value analysis cannot be performed on quantified blot data as this data is non-linear.

### Fluorescence resonance energy transfer (FRET)

Cells were harvested, fixed for 10 min with 2% paraformaldehyde, and permeabilized with ice cold methanol as previously described [34]. Cells were resuspended in 200 μl of PBS containing 2.5 μl of primary rabbit anti-c-Cbl (Santa Cruz, sc-170, C-15), mouse anti-AhR (Abcam, Cambridge, MA), mouse anti-Cbl b (Santa Cruz), rabbit anti-AhR (Santa Cruz) and mouse anti-CD38 (BD Biosciences, San Jose, CA, BD 555458) antibodies and then stained with Alexa 488- and 594-conjugated goat anti-mouse and goat anti-rabbit secondary antibodies, respectively (Invitrogen). The immunocomplexes were analyzed using flow cytometry (BD FACS ARIA III SORP, BD Biosciences). The Alexa 488 emission from 488 nm excitation was collected through a 505 longpass dichroic and 515/20 bandpass filter (channel 488–1). Alexa 594 emission from 532nm excitation was collected through a 600 longpass dichroic and 610/20 bandpass filter (channel 532–2). To measure the FRET signal, a 488 nm laser line was used to excite Alexa 488, which in turn induced Alexa 594 emission. This emission was collected through a 600 longpass dichroic and 610/20 bandpass filter (channel 488–2). Controls with secondary antibody(s) only, secondary(s) plus donor or acceptor primary antibody, and one donor or acceptor primary antibody and the corresponding isotype control for the other antibody plus secondary antibodies were included. Cells stained with just one primary antibody and Alexa 488 and 594 were used for compensation controls for spillover into all fluorescence collection channels. For the c-Cbl+AhR pair, the compensations used were: 488-2 – 532-2 (5.00%), 532-2 – 488-1 (0.70%), 488-2 – 488-1 (9.00%). For the Cbl-b+AhR pair, the compensations used were: 488-2 – 532-2 (6.50%), 532-2 – 488-1 (0.70%), 488-2 – 488-1 (8.00%). For the c-Cbl+CD38 pair, the compensations used were: 488-2 – 532-2 (5.00%), 532-2 – 488-1 (0.70%), 488-2 – 488-1 (6.80%). The statistical analysis was performed using GraphPad Prism (GraphPad software, San Diego, CA). Means of treatment groups were compared using one-way ANOVA with Tukey’s multiple comparisons test. The data represents the means of three repeats ± S.E.M. A p-value of < 0.05 was considered significant.

## Results and Discussion

### FICZ enhances RA-induced inhibition of cell proliferation via G_0_/G_1_ cell cycle arrest

**Fig 1:**
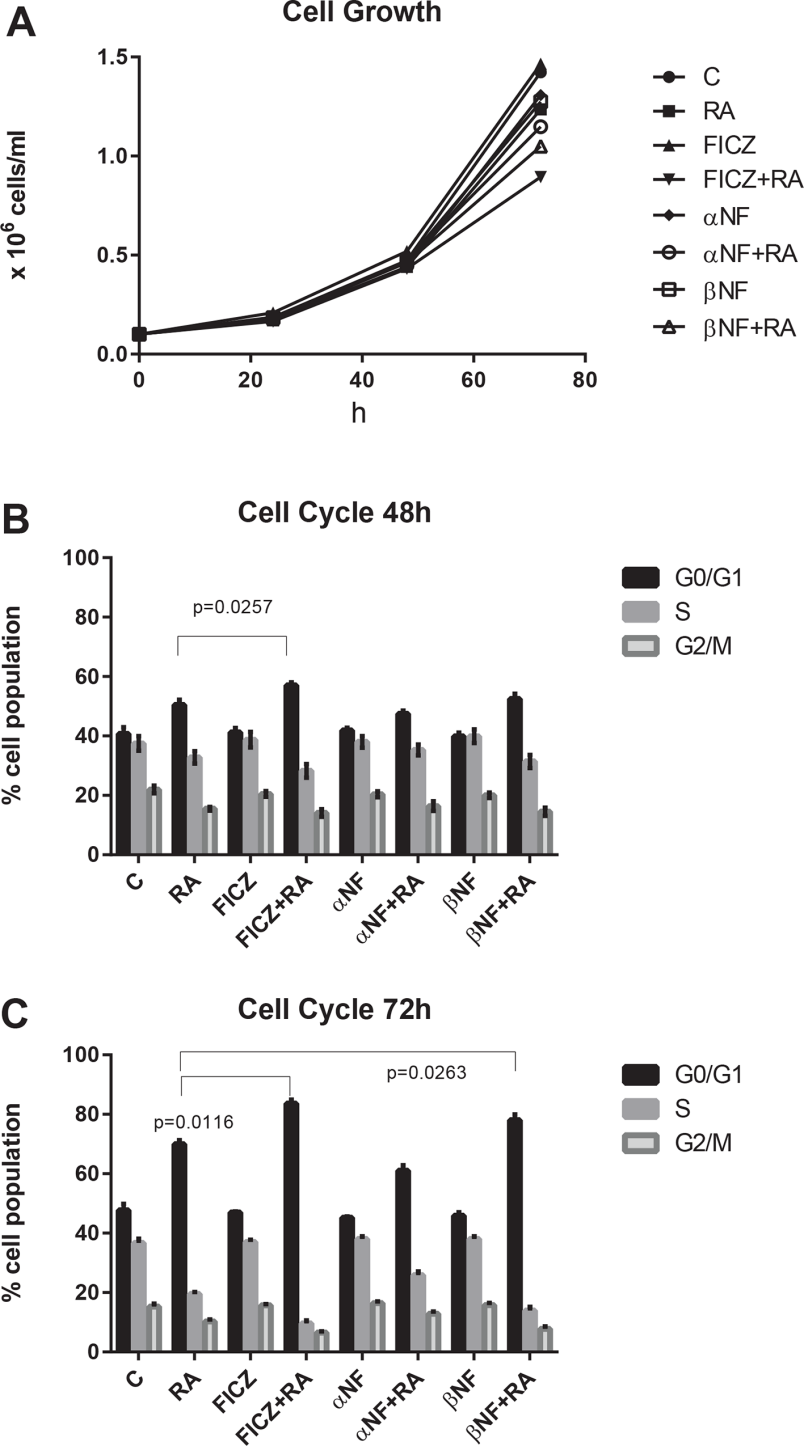
FICZ and RA co-treatment inhibits cell proliferation and augments RA-induced G_0_/G_1_ cell cycle arrest. HL-60 cells were initiated in culture at 0.1 x 10^6^ cells/ml with 1 μM RA, 100 nM FICZ, 1 μM α-NF and 1 μM β-NF as indicated. **(A).** Cells/ml as a function of time in culture. Cell densities in all the cultures remained similar for the first 48 h (averaging among the groups: 0.463±0.01 x 10^6^ cells/ml). By 72 h, the cell population growth is inhibited the most by FICZ+RA (averaging 0.89 x 10^6^ cells/ml), followed by β-NF+RA (averaging 1.05 x 10^6^ cells/ml), compared to RA alone (averaging 1.23 x 10^6^ cells/ml). For untreated cultures cell density was at 1.42 x 10^6^ cells/ml on average. The cell density of FICZ treated cultures was 1.46 x 10^6^ cells/ml on average. **(B).** Percent cells in G_0_/G_1_ after 48 h in culture. After 48 h of culture, cell cycle arrest in G_0_/G_1_ was augmented by FICZ+RA compared to RA alone (p = 0.0257). **(C)**. Percent cells in G_0_/G_1_ after 72 h. in culture After 72 h of culture, FICZ+RA and β-NF+RA induce significantly more G_0_/G_1_ arrest (P<0.0116 and p = 0.0263 respectively) compared to RA alone. AhR modulators used alone had no effect on the cell cycle.

We first determined the effect of FICZ on cell proliferation and cell cycle progression. We also used the AhR agonist (β-NF) and the AhR antagonist (α-NF) to determine if their effects are consistent with FICZ action as an AhR ligand. Cell densities for untreated and treated cultures did not differ greatly for the first 48 h (Fig 1A). By 72 h, cell growth was inhibited the most by combined FICZ+RA, followed by β-NF+RA. In contrast α-NF+RA did not produce as much inhibition as FICZ+RA or β-NF+RA. Interestingly, among all cultures, the greatest apparent proliferation, although not significantly different from control, was that of cells treated with FICZ only showing no toxicity. Cell cycle arrest, evidenced by changes in relative numbers of G_0_/G_1_ cells was consistent with the cell population growth. The cell cycle arrest at 48 h in G_0_/G_1_ was significantly augmented by FICZ+RA compared to RA alone (Fig 1B). After 72 h of culture, both FICZ+RA and β-NF+RA induced significantly higher G_0_/G_1_ arrest compared to RA alone. However, α-NF+RA decreased the G_0_/G_1_ arrest compared to RA only, p = 0.1741 by one-way ANOVA and p = 0.006 comparing by t-test just the RA and α-NF+RA treatments (Fig 1C). All non-RA treated control cases were similar at 48 and 72 h. At 72 h, the untreated cell population has 48% of the cells in G_0_/G_1._ The average percentage of RA treated cells in G_0_/G_1_ at 72 h was 70% and of the RA+FICZ treated cells was 84%. In conclusion, AhR ligands used alone had no effect on cell cycle, but combined with RA the agonists augmented the G_0_/G_1_ arrest while the antagonist diminished it. The results are consistent with the suggestion that the enhancement of RA-induced cell cycle arrest by FICZ is partially mediated by AhR.

### FICZ enhances RA-induced expression of differentiation markers CD38 and CD11b

**Fig 2:**
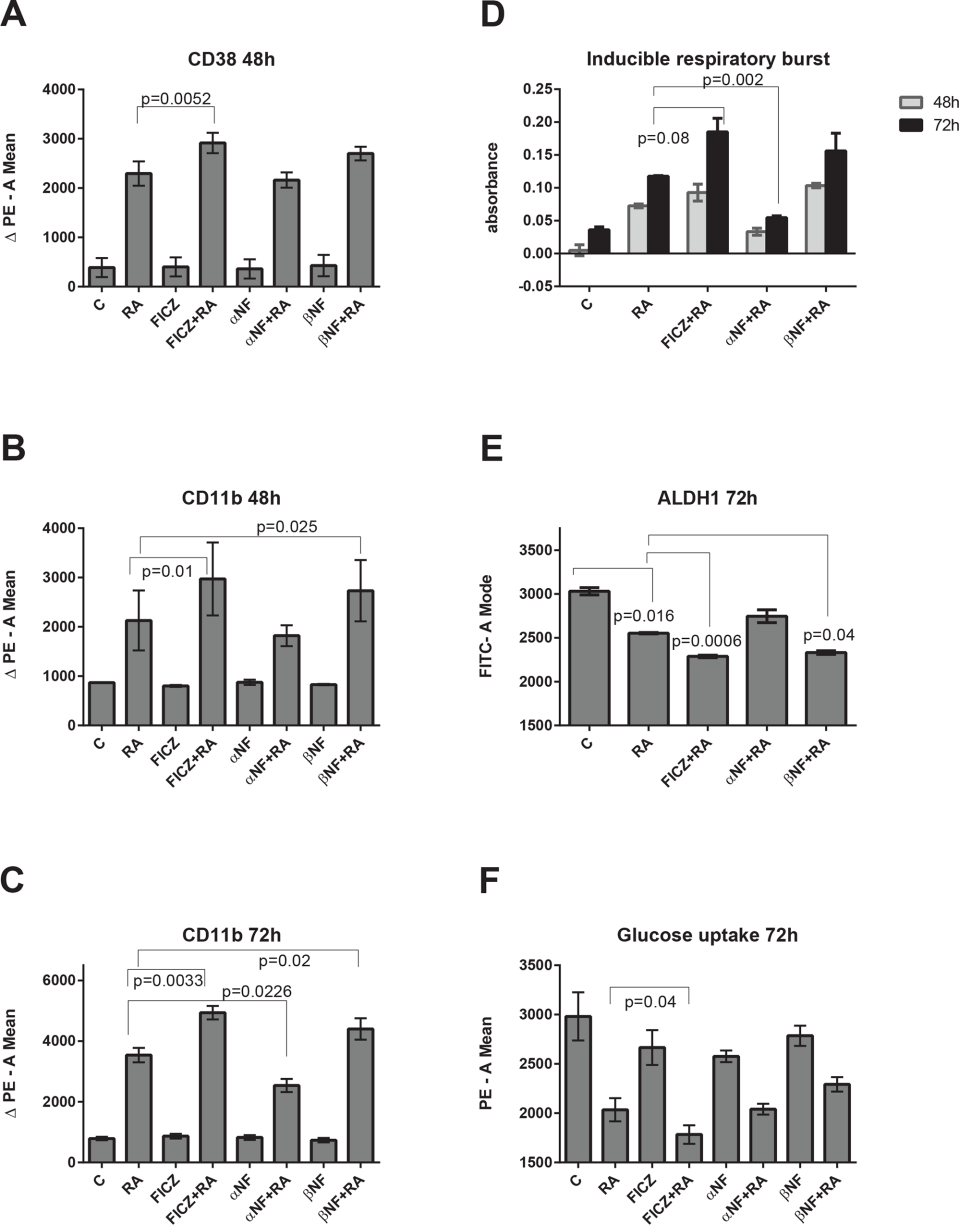
FICZ enhances RA-induced expression of differentiation markers and blunts the stem cell like/malignant markers. HL-60 cells were initiated in culture at 0.1 x 10^6^ cells/ml with 1 μM RA, 100 nM FICZ, 1 μM α-NF and 1 μM β-NF as indicated. Induced differentiation markers were measured at 48 and 72 h by flow cytometry. The results are expressed as mean integral of the PE voltage pulse signal of antibody labeled cells minus the mean integral PE signal of the unlabeled cells (thereby subtracting background signal). **(A).** RA-induced CD38 expression was enhanced by co-treatments of FICZ+RA (mean 2915) and β-NF+RA (mean 2700), and decreased by α-NF+RA (mean 2163) compared to RA (mean 2295). Statistical significance (p = 0.0052) was demonstrable only for FICZ+RA versus RA. **(B).** CD11b expression at 48 h was increased by combining RA with an AhR agonist (FICZ, p = 0.025, or β-NF, p = 0.01) compared to RA alone and decreased (although this did not reach a significance level) by the AhR antagonist α-NF, compared to RA alone. **(C).** CD11b expression at 72 h was increased by combining RA with FICZ, p<0.0033, or with β-NF, p<0.02) and was decreased by the AhR antagonist α-NF in combination with RA, p = 0.0226, compared to RA alone. The FICZ, α-NF and β-NF without RA did not induce CD38, or CD11b expression, compared with untreated control cells. **(D)** FICZ with RA enhances the functional differentiation marker inducible respiratory burst. HL-60 cells were initiated in culture at 0.1 x 10^6^ cells/ml with 1 μM RA, 100 nM FICZ, 1 μM α-NF and 1 μM β-NF as indicated. TPA induced respiratory burst was measured at 48 and 72 h by an NBT reduction assay. Compared to RA alone, FICZ+RA enhances respiratory burst (p = 0.08 at 72 h) and α-NF+RA decreases it (P = 0.002 at 72 h). **(E)** FICZ enhances the RA-induced decreased expression of the stem cell marker, ALDH1. HL-60 cells were initiated in culture at 0.1 x 10^6^ cells/ml with 1 μM RA, 100 nM FICZ, 1 μM α-NF and 1 μM β-NF as indicated. Flow cytometric analysis of live cells was performed setting the logical gate to exclude 95% of the untreated cells. Aldehyde dehydrogenase1 enzymatic activity is significantly lower (p = 0.0160) in RA-treated cells than in untreated control cells. FICZ+RA, and β-NF+RA, have lower ALDH1 (p = 0.0006 and p = 0.04 respectively) compared to RA alone. **(F)** FICZ enhances RA-induced reduction of glucose uptake. HL-60 cells were initiated in culture at 0.1 x 10^6^ cells/ml with 1 μM RA, 100 nM FICZ, 1 μM α-NF and 1 μM β-NF as indicated for 72 h. All the cells treated with RA had significantly less glucose uptake, compared to untreated cells, p = 0.03. FICZ+RA cells had significantly less glucose uptake than the cells treated with RA only (p = 0.04).

We previously reported that FICZ with RA enhances CD11b expression compared to the expression induced by RA alone [53]. Here we investigated the effects of the established AhR antagonist and agonist, α-NF and β-NF to compare them with the effects of FICZ. As shown in Fig 2, the expressions of CD38 and CD11b membrane bound differentiation markers are modulated by AhR ligands during RA-induced differentiation, but are not affected by the AhR ligands alone. FICZ enhances the expression of RA-induced CD38 expression at 48 h, while α-NF and β-NF do not have a statistically significant effect on CD38 (Fig 2A). CD11b expression, at both 48 and 72 h (Fig 2B and 2C), is also significantly augmented by FICZ+RA compared to RA alone. β -NF also significantly enhances RA-induced CD11b expression (48 and 72 h), whereas α -NF significantly reduces CD11b expression compared to RA alone (72 h). Again AhR agonists and an antagonist elicit cellular responses consistent with respectively positive and negative regulation of RA-induced differentiation.

### FICZ enhances RA-induced functional differentiation marker, inducible respiratory burst

Induced respiratory burst, an innate myeloid cell response upon exposure TPA and a functional marker of granulocytic maturity, was measured at 48 and 72 h by reduction of nitroblue tetrazolium (NBT). FICZ augments the RA-induced respiratory burst compared to RA alone at 72 h (Fig 2D). In contrast, the antagonist α-NF significantly reduces the RA-induced respiratory burst at 72 h. β-NF tends to increase respiratory burst, but the measured change did not achieve statistical significance.

### Co-treatment with FICZ and RA decreases the stem cell marker ALDH1

Aldehyde dehydrogenase 1 (ALDH1) enzymatic activity is a marker for normal and cancer stem cells. The effects of RA alone and in combination with FICZ, β–NF and α–NF on this stem cell marker were determined. ALDH1 activity is significantly reduced in RA-treated HL-60 cells compared to untreated control cells (Fig 2E). Combined FICZ+RA or β-NF+RA treatment further lowered ALDH1 activity compared to RA alone. This suggests that the AhR agonists promote RA-induced differentiation toward a more functionally mature state. The AhR antagonist α-NF, however, tended to counter the effects of RA, resulting in an increase of ALDH1 activity, though the measurement did not reach statistical significance. The results for ALDH1 thus support the above results for growth and differentiation regulated by AhR agonists or antagonist.

### RA diminishes cellular glucose uptake which is further reduced by adding FICZ

Increased glucose uptake is a tumor marker associated with aerobic glycolysis, the Warburg effect. To determine if RA and the AhR ligands regulate loss of this tumor attribute, cultures were treated for 48 h and glucose uptake measured. All cells treated with RA exhibited significantly less glucose uptake, compared to untreated cells (Fig 2F). For FICZ+RA cells, uptake was significantly less than for cells treated with RA only. However, α-NF and β-NF did not modulate glucose uptake. Nevertheless, the FICZ effect is of potential therapeutic significance since FICZ is endogenous, i.e. part of the metabolic profile [57, 58], and glucose metabolism has been proposed as a marker of risk stratification in AML [59]. Moreover, it was reported that c-Cbl Y371H in AML samples correlates with increased cellular glucose metabolism [60].

### Clustering analysis verifies differentiation markers and inducers

**Fig 3:**
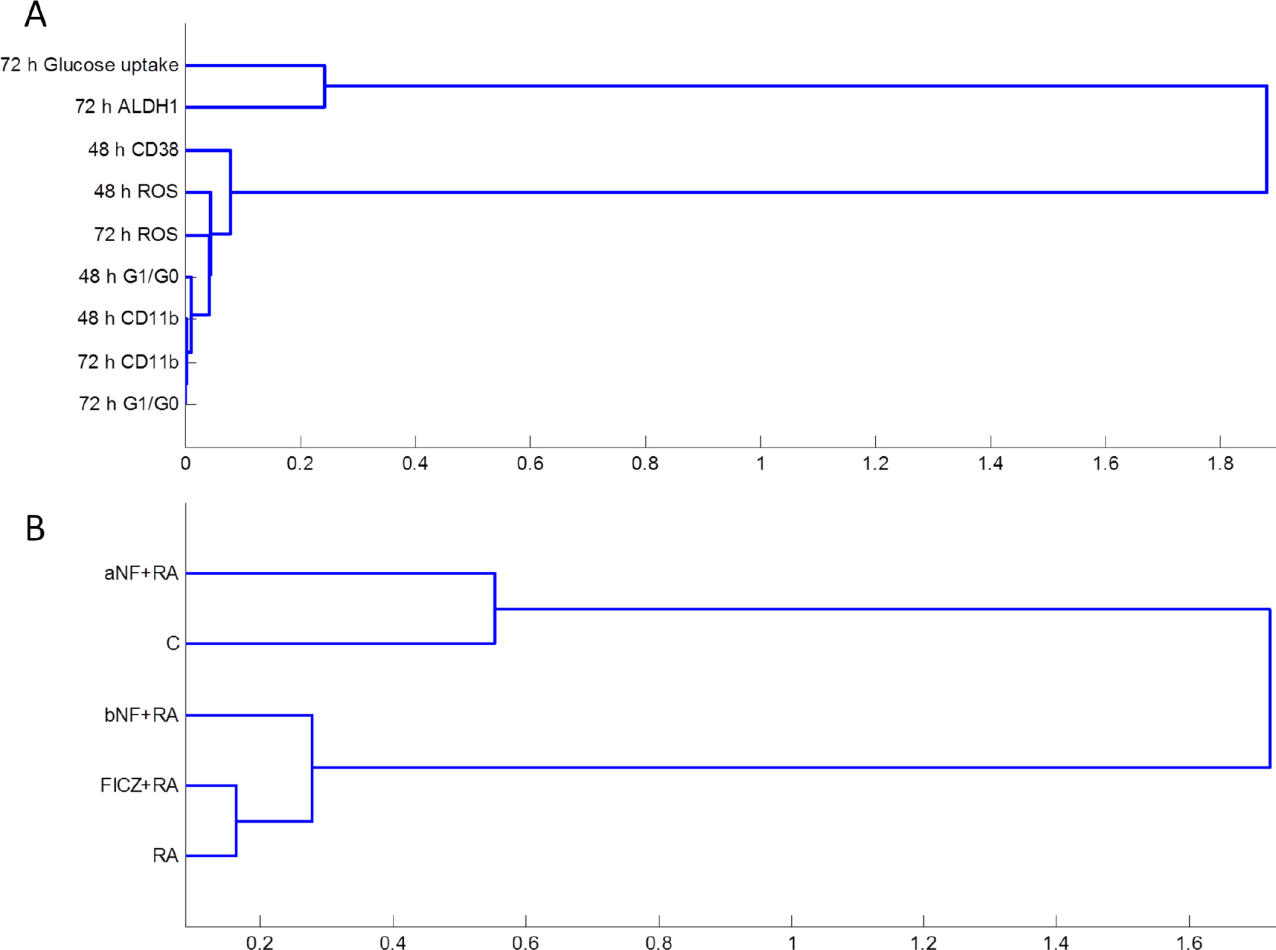
Hierarchical clustering between phenotypic markers (A) and treatments (B). The phenotype data was subject to hierarchical clustering analysis using the Pearson correlation coefficient as a distance metric and the average linkage method. Distances between clusters (1-Pearson correlation coefficient) are indicated on the x-axis.

Based on the phenotypic data for the treatments, we performed a clustering analysis of the differentiation markers. The stem-like and malignancy markers ALDH and glucose uptake were the least correlated to the differentiation markers, as expected, since these markers were diminished upon RA treatment whereas the others were enhanced. The other phenotypic markers, CD38, CD11b, G_0_/G_1_ arrest and respiratory burst (ROS), presented a closer similarity based on time point rather than on the specific differentiation marker, with the exception of the late differentiation marker ROS which clustered close to the early differentiation marker CD38 (Fig 3A). Next, clustering analysis was performed based on main treatments for all the differentiation markers (Fig 3B); α-NF+RA clustered closely with untreated control, which were far removed from successful inducers of differentiation. Among those, it is interesting that FICZ+RA clustered closer to RA than to β-NF+RA, as another example among many examples recently published by other groups highlighting the differences in biological responses between AhR agonists [61, 62].

### FICZ acts as an AhR ligand upregulating Cyp1A1 expression

**Fig 4:**
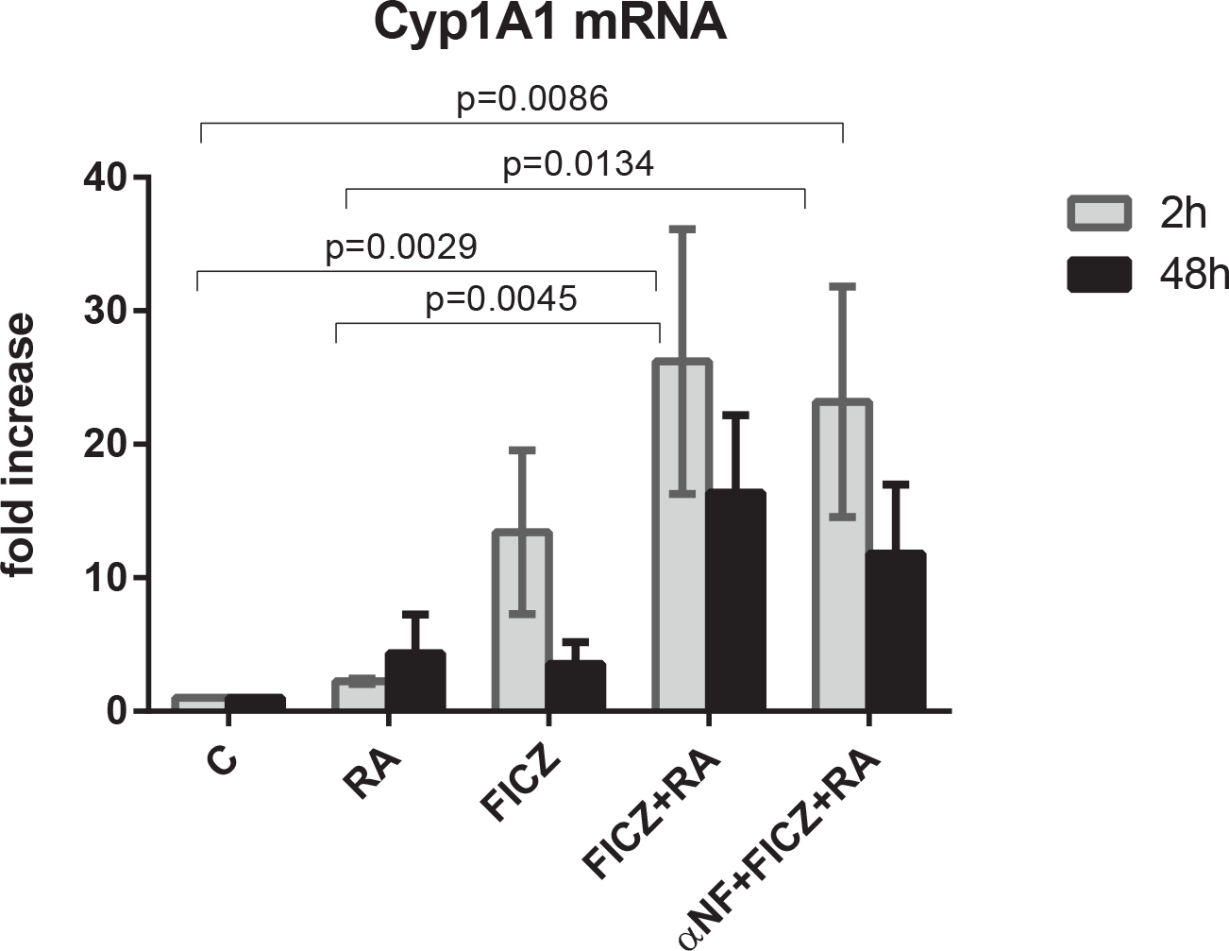
FICZ upregulates Cyp1A1 expression. HL-60 cells were initiated in culture at 0.1 x 10^6^ cells/ml with 1 μM RA, 100 nM FICZ, 1 μM α-NF as indicated for 2 h and 48 h. At 2 h post treatment, FICZ upregulates Cyp1A1 expression in HL-60 cells, reaching significance for RA+FICZ vs C (p = 0.0029) and vs RA (p = 0.0045) and α-NF+FICZ+RA vs C (p = 0.0086) and vs RA (p = 0.0134).

In order to confirm the increase of AhR transcriptional activity induced by FICZ, we assayed the expression level of Cyp1A1 at 2 h and 48 h post treatment by real-time PCR. At 2 h, FICZ increased Cyp1A1 expression by an average of 10 fold, while FICZ and RA significantly increased Cyp1A1 expression both as compared to untreated control and to RA-treated cases (Fig 4). The addition of the antagonist α-NF to FICZ+RA treatment slightly reduced the expression of Cyp1A1 compared to FICZ+RA only. At 48 h, the expression of Cyp1A1 was diminished in all treatment cases, and was not significantly different than the expression in untreated cells at this time point. This suggests that, although at early time points FICZ induces AhR transcriptional activity, at later time points this activity is not a significant factor for the observed effects. This is consistent with reports that FICZ is metabolized [63] as well as with reports that, at later time points post RA treatment, AhR is primarily localized in the cytosol [29]. We therefore next investigated if FICZ+RA treatment affects the signalsome known to drive RA-induced differentiation.

### FICZ modulates an RA-elicited signalsome

RA-induced differentiation is driven by a signalsome that includes MAPK signaling molecules and regulators thereof, such as c-Cbl and SFKs. To identify AhR directed components of the signalsome, we determined how FICZ, α-NF and β-NF regulated signalsome constituents and their interactions. Immunoblots of whole cell lysates were used to assess the expression and activation of known signaling proteins implicated during RA-induced differentiation as well as previously unexplored proteins (Cbl-b, GSK3, p38α, PU.1, MAFB, ARNT). The known signalsome proteins include c-Raf and its S621 and S259 site specific phosphorylation sites, Src family kinases Lyn and Fgr, CD38-associated factors c-Cbl and Slp76, and AhR, which are all upregulated and/or activated during RA-induced differentiation in HL-60 cells. Although c-Raf is the apex of the Raf/MEK/ERK signaling cascade, c-Raf may exhibit noncanonical and ERK-independent functions during RA-induced differentiation. pS621c-Raf is localized in the nucleus of RA-treated HL-60 cells [64] and more specifically is colocalized with NFATc3 on the BLR1 gene promoter [65]. Src family kinases (SFKs) are known to regulate the MAPK pathway [66–69]. The predominant SFKs in myeloid cells are Lyn and Fgr, with Lyn exhibiting RA-induced phosphorylation at the SFK activation site Y416 (Lyn Y397) while Fgr is not phosphorylated [22]. Lyn and Fgr have been shown to exist in distinct membrane compartments, suggesting divergent signaling roles [70]. We have also shown that Lyn interacts with pS259c-Raf [22].

Previously we reported that FICZ indeed enhances the RA-induced phosphorylation of c-Raf at S621 and S289/296/301, and enhances expression of Fgr, c-Cbl and AhR [53]. We also showed that the AhR agonist β-NF can augment RA-induced upregulation of pS621c-Raf, Fgr, Lyn and Lyn phosphorylation, while α-NF treated cells failed to upregulate these factors [53]. Among those proteins forming the RA-induced signalsome, c-Cbl is of particular interest. It is frequently mutated in juvenile myelomonocytic leukemia [71]. It is upregulated by RA when the leukemic cells are induced to differentiate, and this is enhanced by FICZ co-treatment. c-Cbl is downstream of CD38, and other groups had reported a Fgr-Cbl interaction [72]. This motivated interest in determining if the enhanced RA-induced differentiation attributed to AhR agonists reflected regulation these putative signalsome components and their interaction. Accordingly we treated cells with the agents, harvested cellular lysate after 48 h and analyzed expression and associations of these signalsome entities with western blotting and flow cytometry FRET.

**Fig 5:**
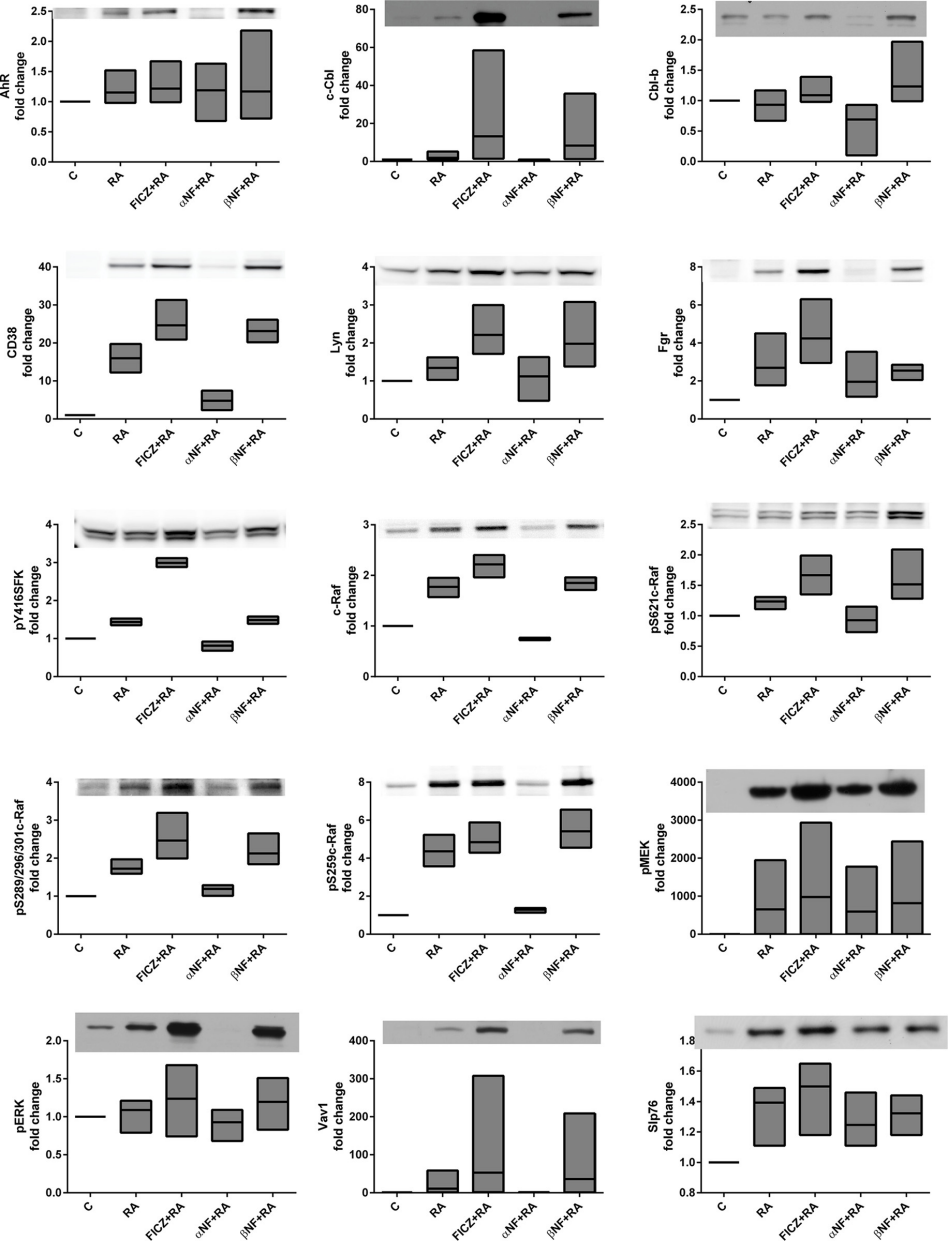
FICZ modulates RA-elicited signalsome. HL-60 cells were initiated in culture at 0.1 x 10^6^ cells/ml with 1 μM RA, 100 nM FICZ, 1 μM α-NF and 1 μM β-NF as indicated. Western blot assay of whole cell lysates is shown for AhR, c-Cbl, Cbl-b, CD38, Lyn, Fgr, pY416SFK, c-Raf, pS621c-Raf, pS289/296/301c-Raf, pS259c-Raf, pMEK, pERK, Vav1, Slp76. Whole cell lysates were collected after 48 h. Lysates were resolved on a 12% polyacrylamide gel. 25 μg of protein was loaded in each well. Experiments were repeated at least three times. All the western blot data were quantified using ImageJ, normalized to GAPDH and then to untreated control and graphed as min to max floating bars with a line at the mean using GraphPad Prism software. P-value analysis cannot be performed on quantified blot data as this data is non-linear. A representative blot is shown for each marker.

**Fig 6:**
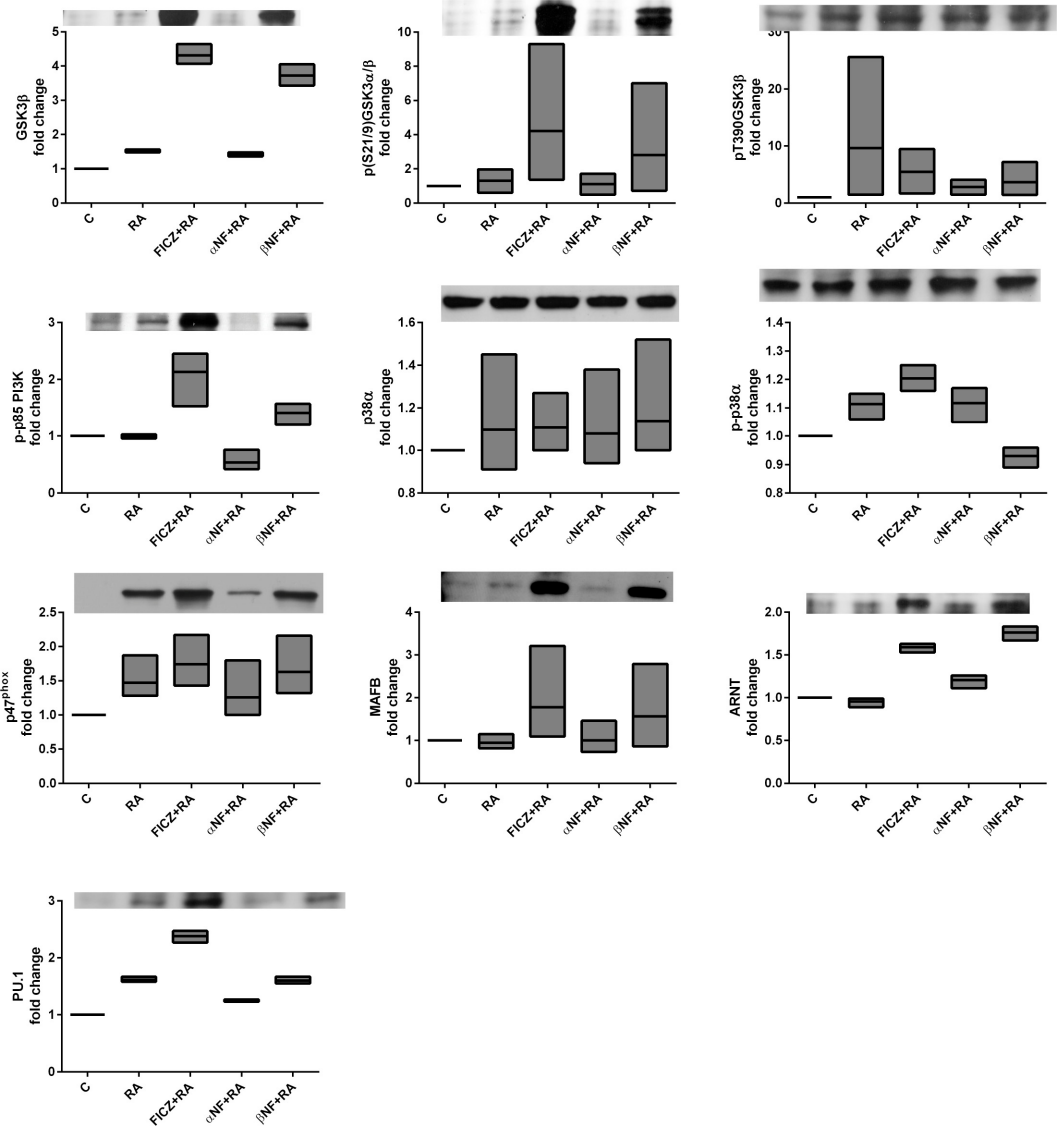
FICZ modulates RA-elicited signalsome. HL-60 cells were cultured and treated as described in Fig 5. Western blots for GSK3β, p(S21/9)GSK3α/β pT390GSK3β, p-p85 PI3K, p38α, p-p38α, p47^phox^, MAFB, ARNT, and PU.1 was performed as described in Fig 5.

**Fig 7:**
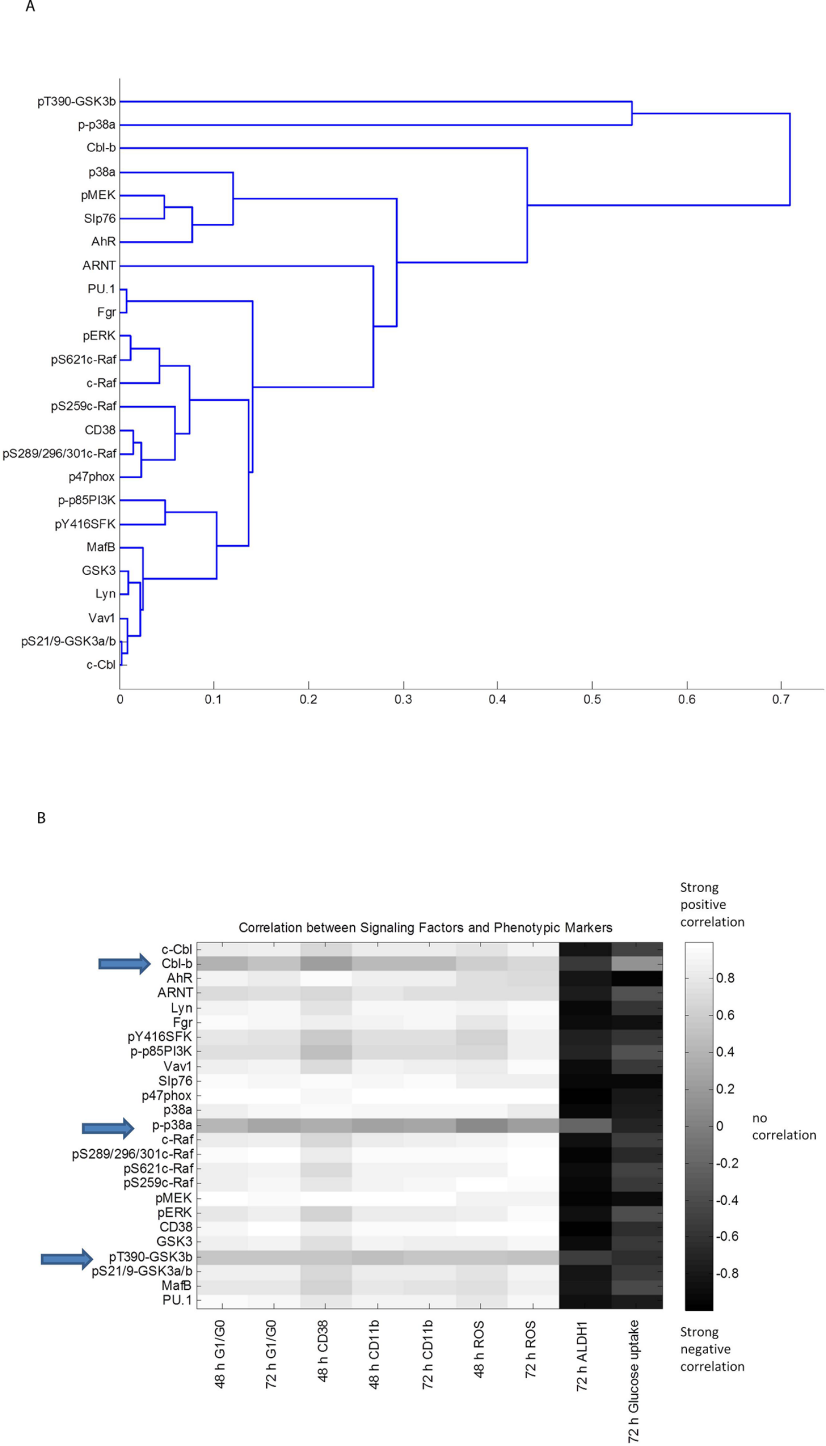
Correlation analysis of the FICZ + RA-elicited signalsome. (A) The dendrogram resultant from the hierarchical clustering analysis of the data presented in Fig 5 using the Pearson correlation coefficient as the distance metric and the average linkage method. Distances between clusters (1-Pearson correlation coefficient) are indicated on the x-axis. (B) Pearson correlation coefficient matrix between signaling molecules and phenotypic markers for the control, RA, FICZ+RA, α-NF +RA and β-NF +RA.

We first confirmed that the two AhR ligand agonists, FICZ and β-NF, can enhance the RA-induced expression of signalsome components, the most prominent being CD38, c-Cbl, and Fgr (Fig 5). In contrast, the antagonist α-NF prevents the RA-induced increase of CD38 and c-Cbl, expression. α-NF also reduced levels of c-Raf expression and S259 and S621 phosphorylation, whereas the agonists maintained or enhanced these effects. The aforementioned proteins, in addition to pY416phospho-activated SFKs and pS289/296/301 (C-terminal domain) c-Raf, appear closely coupled based on their responses to 48 h RA and AhR agonist/antagonist treatments. This suggests they are implicated in the CD38-associated signaling cascade responsible for RA-induced differentiation (Figs 5 and 6). Clustering analysis of the western blot data for all the repeats confirms the tight association of those signalsome components: c-Raf, pERK, CD38, PI3K, pY416SFK, Lyn, Vav1, c-Cbl (Fig 7A). pT390-GSK3β and p-p38α are the least associated with the rest of the proteins surveyed, followed by Cbl-b. As these proteins are distant (using 1-Pearson correlation coefficients as the distance metric between cluster elements) from the other proteins surveyed, t they may not regulate RA-induced differentiation. AhR and ARNT are in separate subclusters suggesting a non-transcriptional role for AhR in the RA-induced signalsome.

**Fig 8:**
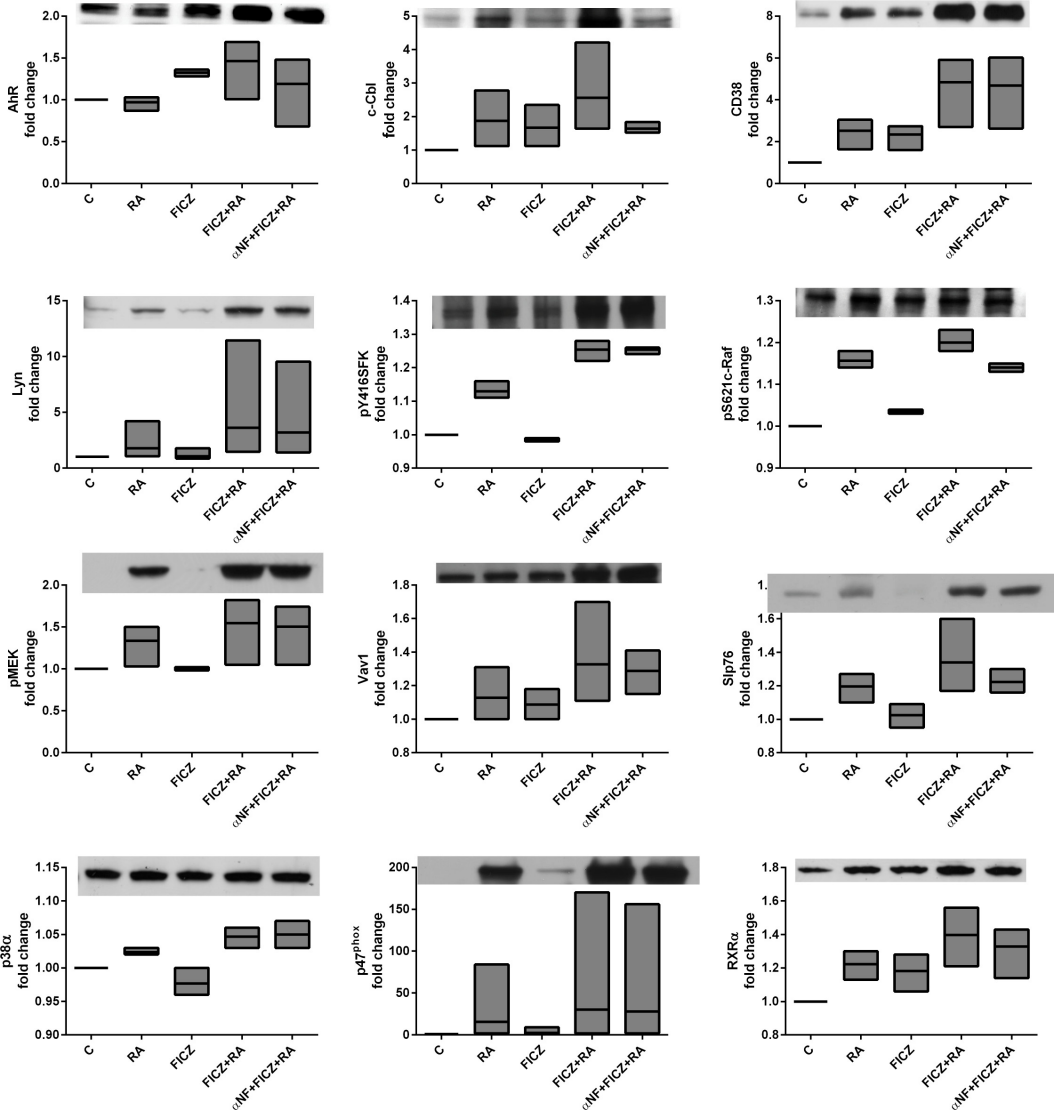
FICZ has effects beyond classical AhR transcriptional regulation. HL-60 cells were initiated in culture at 0.1 x 10^6^ cells/ml with 1 μM RA, 100 nM FICZ, 1 μM α-NF and 1 μM β-NF as indicated. Western blot assays of whole cell lysates are shown. Whole cell lysates were collected after 48 h. Lysates were resolved on a 12% polyacrylamide gel. 25 μg of protein was loaded in each well. Experiments were repeated at least three times. All the western blot data were quantified using ImageJ, normalized to GAPDH and then to untreated control and graphed as min to max floating bars with a line at the mean using GraphPad Prism software. P-value analysis cannot be performed on quantified blot data as this data is non-linear. A representative blot is shown for each marker.

Next, we calculated Pearson correlation coefficients between quantified repeat western blot data and phenotypic markers to generate a heat map (Fig 7B). There is again a very strong positive correlation between the phenotypic markers and CD38, MAPK(Raf/MEK/ERK) axis, SFKs, AhR and c-Cbl. Cbl-b, p-p38α and pT390-GSK3β are not correlated with the differentiation markers, suggesting they are not part of the signalsome. For a selected ensemble of markers, additional experiments were done to assess the effect of FICZ, FICZ+RA, or α-NF treated with FICZ+RA (Fig 8). These results indicate that not all FICZ+RA effects are due to AhR classical transcriptional activity known to be upregulated by FICZ and downregulated by α-NF. For example, pMEK is upregulated by RA but not by FICZ, yet FICZ enhances RA-induced upregulation of pMEK. When α-NF is added to FICZ+RA, pMEK abundance is not diminished. Other transcription factors likely involved in the FICZ-enhanced RA-induced transcriptional upregulation of signalsome members include PU.1 and MAFB (Fig 6) and RXRα (Fig 8). These transcription factors are upregulated by FICZ+RA compared to RA indicating effects of FICZ+RA beyond simply AhR regulated transcription.

### FICZ induces a c-Cbl-AhR association in RA-treated samples

**Fig 9:**
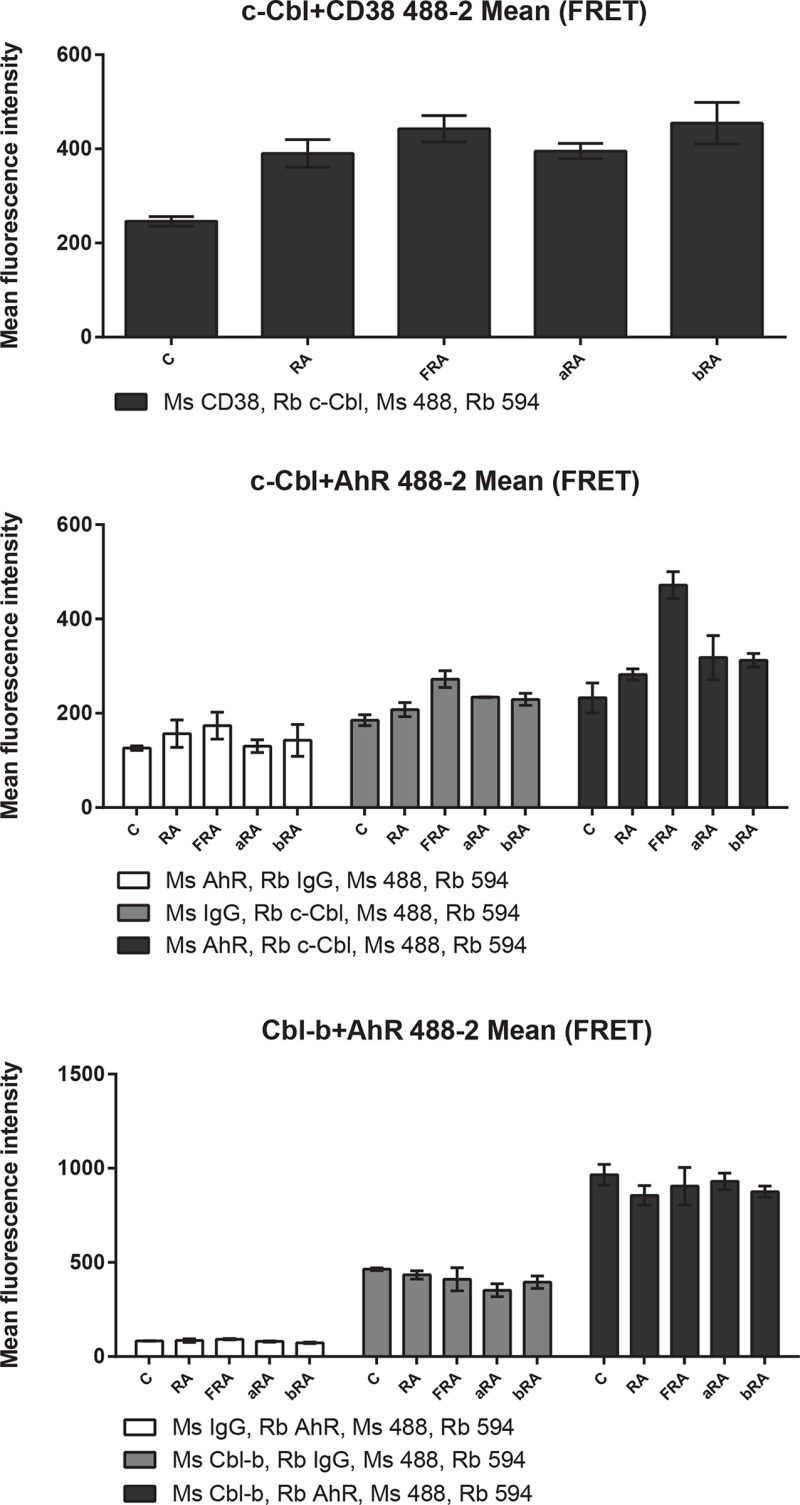
FICZ induces a c-Cbl-AhR association (mean fluorescence). HL-60 cells were initiated in culture at 0.1 x 10^6^ cells/ml with 1 μM RA, 100 nM FICZ, 1 μM α-NF and 1 μM β-NF as indicated. Cells were harvested, fixed for 10 min with 2% paraformaldehyde, and permeabilized with ice cold methanol. Cells were labeled with the primary antibodies (or isotype controls) and then stained with Alexa 488- and 594-conjugated goat anti-mouse and goat anti-rabbit secondary antibodies, respectively (Invitrogen). The immunocomplexes were analyzed using flow cytometry (BD FACS ARIA III SORP, BD Biosciences). Mean fluorescence intensity is shown.

**Fig 10:**
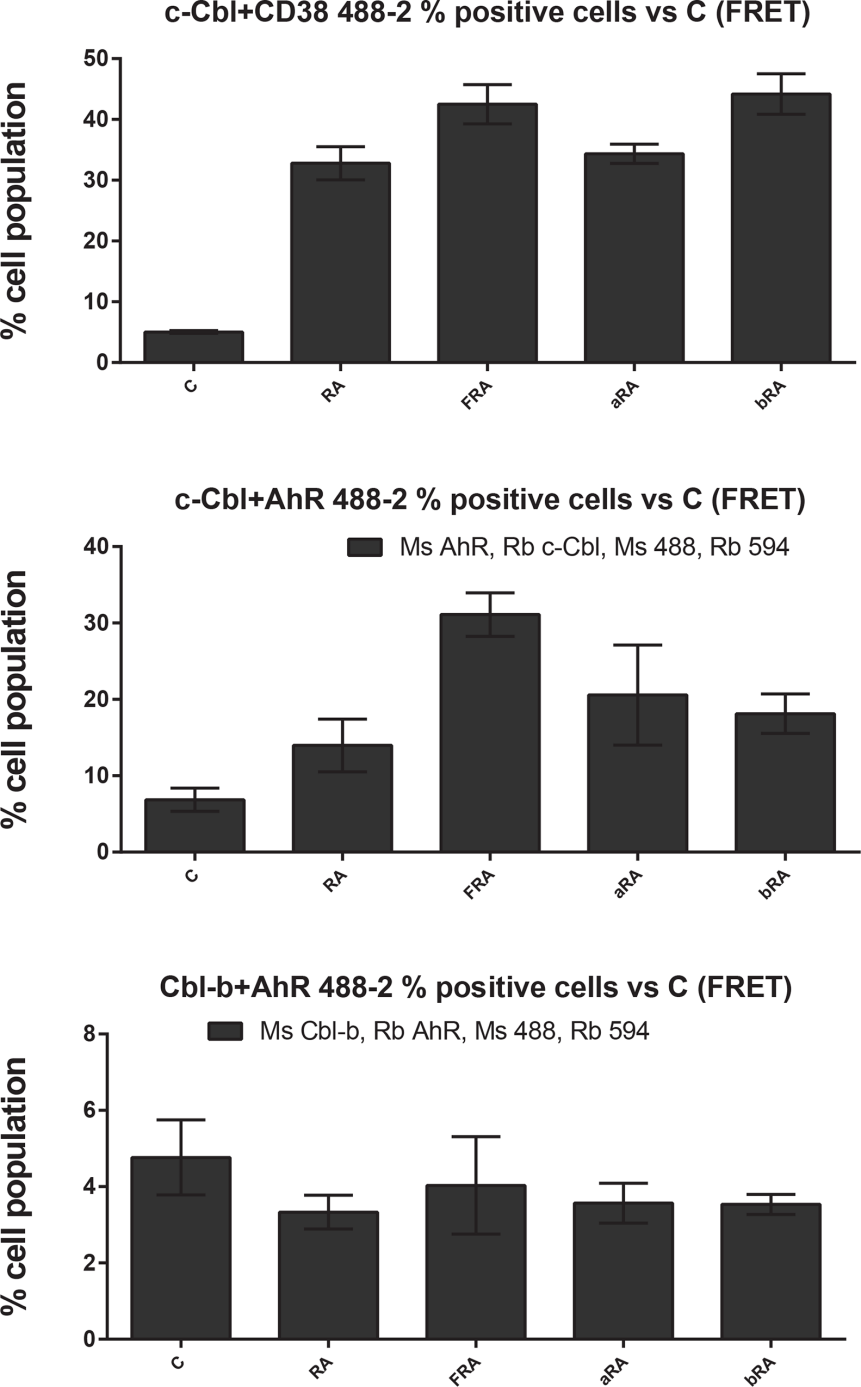
FICZ induces a c-Cbl-AhR association (percentage of positive cells). Percentage of positive cells, setting the control at 5% positive, from data described in Fig 9 is shown.

Since both c-Cbl and AhR were present in the signalsome and their expression levels are modulated by AhR ligands, we assessed the c-Cbl-AhR association using fluorescence resonance energy transfer (FRET) in fixed cells measured by flow cytometry (Figs 9 and 10 and S1–S3 Figs). FRET is a distance-dependent transfer of energy from the electronic excited state of a donor fluorochrome to an acceptor fluorochrome by non-radiative dipole-dipole coupling. Excitation of the donor thus results in fluorescence emission by the acceptor. The efficiency of FRET is dependent on the inverse sixth power of the intermolecular separation: E = 1/[1+(r+R_0_)^6^], where r is the distance between the donor and acceptor and R_0_ is the Forster distance, the distance at which the E is 50% for this donor-acceptor pair. The R_0_ for Alexa Fluor 488 and Alexa Fluor 594 is 60 Å (www.lifetechnologies.com).

First, we assessed the effect of AhR agonists and antagonist on CD38-c-Cbl interaction, an RA-induced interaction that, when disrupted, results in diminished differentiation [23, 34]. The AhR ligands did not modulate the RA-induced interaction between CD38 and c-Cbl by mean fluorescence intensity (Fig 9 and S1 Fig) or as percent positive cells (Fig 10 and S1 Fig).

Next, we assessed the interaction between c-Cbl and AhR. Although the mean fluorescence intensity was not increased by RA, it was increased by FICZ+RA (Fig 9). The percent positive cells were increased compared to control only in the samples treated with RA plus an AhR agonist (p = 0.0101 for RA+FICZ and p = 0.0487 for samples treated with RA+ β-NF, Fig 10 and S2 Fig). Based on clustering analysis presented earlier, Cbl-b is not correlated with the RA-induced signalsome. However, we also assessed the association of Cbl-b with AhR by flow cytometry FRET. Cbl-b has different functions than c-Cbl and participates in distinct protein complexes as reported for other cell types [73]. In HL-60 cells, Cbl-b and AhR are constitutively associated (Figs 9 and 10 and S3 Fig).

**Fig 11:**
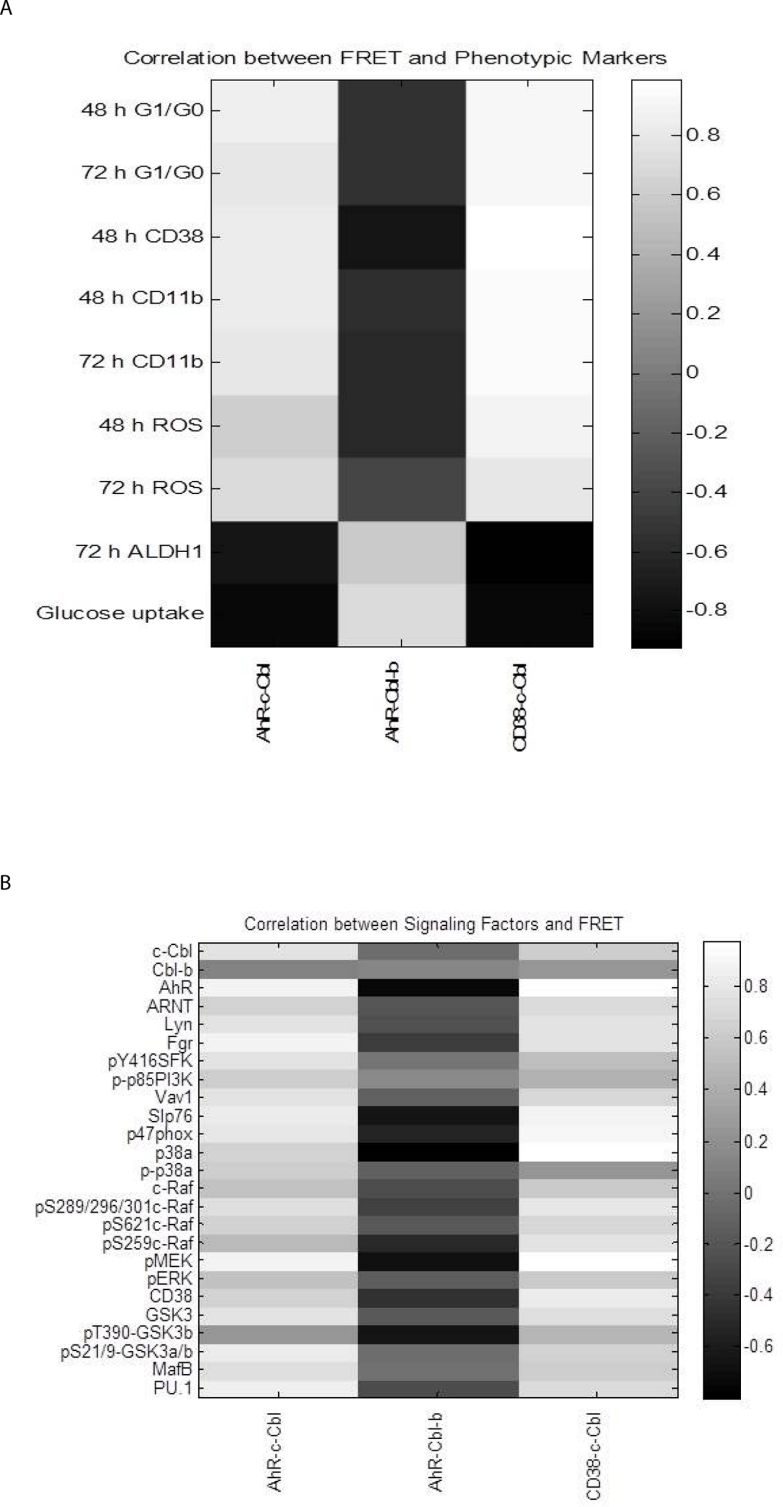
Correlation analysis of the protein-protein associations and (A) differentiation markers and (B) signaling molecules.

We generated a Pearson correlation coefficient matrix between differentiation markers and FRET results across all treatments (Fig 11A). CD38 and CD11b differentiation markers very strongly correlate with the associations between c-Cbl and both CD38 and AhR. This is consistent with the notion that CD38, c-Cbl, and AhR are functioning closely together at the plasma membrane pointing to early dependence. Finally, a Pearson correlation coefficient matrix was generated between the signaling molecules and FRET results (Fig 11B) show a positive correlation with AhR-c-Cbl interaction and SFKs, the Raf/MEK/ERK axis, Vav1, Slp76, pS21/9-GSK3α/β, MAFB and PU.1.

## Conclusions

Taken together, our results show that addition of FICZ enhances RA-induced differentiation by AhR-dependent signaling effects as well as potential AhR-independent effects. Moreover, FICZ and RA co-treatment also employ some protein associations that are not elicited by RA alone. This potentially has significant translational implications for the extension of RA induction therapy, using cotreatment with FICZ, to malignancies currently just marginally responsive to RA by recruiting additional pathways.

## Supporting Information

S1 Fig c-Cbl-CD38 association. Fluorescence histograms of data described in Fig 9 are shown.
(EPS)

S2 Fig FICZ induces a c-Cbl-AhR association. Fluorescence histograms of data described in Fig 9 are shown. Left and middle columns: fluorescence histograms of FRET controls with rabbit (Rb) or mouse (Ms) isotype controls. Right column: fluorescence histogram of FRET.
(EPS)

S3 Fig Cbl-b-AhR association. Fluorescence histograms of data described in Fig 9 are shown. Left and middle columns: fluorescence histograms of FRET controls with rabbit (Rb) or mouse (Ms) isotype controls. Right column: fluorescence histogram of FRET.
(EPS)
